# Prevalence of Self-Reported Symptoms of Sexually Transmitted Infection among Establishment-Based Female Sex Workers in Ethiopia

**DOI:** 10.1155/2020/8848016

**Published:** 2020-12-14

**Authors:** Shewangizaw Hailemariam, Aderajew Nigusse, Alemi Kebede

**Affiliations:** ^1^Department of Midwifery, College of Health Science, Mizan-Tepi University, Mizan-Teferi, Ethiopia; ^2^Department of Population and Family Health, Institute of Health Science, Jimma University, Jimma, Ethiopia

## Abstract

**Introduction:**

In spite of the fact that female sex workers being regarded as core transmitters of sexually transmitted infections to the general population, previous studies undertaken around STIs in Ethiopia fail to consider this segment of the population. Hence, the present study investigated the prevalence of self-reported symptoms of STIs and the risk factors among FSWs in Adama Town, Ethiopia.

**Method:**

A cross-sectional study was conducted from May 01, 2017, to April 30, 2017, in Adama Town. Three hundred ninety female sex workers were selected from 120 licensed drinking establishments by using simple random sampling technique. The interviewer-administered structured tool was used to collect data. Data were entered using EPI data version 3.1 and exported to SPSS version 20 for analysis. Bivariate and multivariable logistic regression analyses were used to identify factors associated with the outcome variable.

**Result:**

Among the requested 423 female sex workers, 390 willingly responded to the interviewer-administered structured questionnaires making a response rate of 92.2%. One hundred thirty-nine (35.6%, 95% CI (33.8%–37.4%) of the female sex workers reported one or more STI symptoms. Several risk factors were identified including inconsistent condom use with a nonpaying client (AOR = 5.43, 95% CI: 2.73, 10.80), alcohol use before sex (AOR = 2.41, 95% CI: 1.35, 4.30), longer duration of sex work (AOR = 2.27, 95% CI: 1.26, 4.08), and having poor knowledge of STIs (AOR = 2.44, 95% CI: 1.31, 4.54).

**Conclusion:**

Self-reported symptom of STI among female sex workers in Adama Town is relatively high when compared with previous studies. Hence, health education programs should address the issue of inconsistent condom use with nonpaying clients, alcohol consumption before sex, and knowledge of STIs, with a particular emphasis on those female sex workers who stayed longer in the business.

## 1. Introduction

Sexually transmitted infections (STIs) refer to those conditions caused by pathogens that can be acquired and transmitted through unprotected sexual intercourse. They can be caused by bacteria, viruses, protozoa, fungi, and ectoparasites [1, 2]. STIs are among the most common cause of illness in the world and have far-reaching health, social, and economic consequences. They have public health importance because of their magnitude, potential complications, and their interaction with HIV/AIDS [2].

Female sex workers (FSWs) have been recognized in Ethiopia since olden times although there are no documented data as to when and where FSWs first appeared in the country. Few sources link the beginnings of sex business with the movement of kings, nobles, and warlords, the development of cities, and the advancement of trading [3]. In Ethiopia, it is illegal to operate a brothel establishment or procure sex workers for commercial purposes, but the sale of sex by women is not prohibited by law [4]. Due to their high HIV prevalence, their increased ability to transmit HIV when coinfected with other STIs, and to the larger group they reach through their sexual network, sex workers have long been targeted as the “core group” most at risk of acquiring STI and HIV [5].

The number of women entering in sex business is increasing especially in the sub-Saharan African countries escalated by problems associated with urbanization, pressure from poverty, food and political insecurity, and civil unrest [6, 7]. In many societies, FSWs face stigmatization, marginalization, and discrimination even in the health-care sector, which make the prevention and control of STI and HIV challenging [8, 9]. Due to this fact, FSWs tend to self-diagnose and procure over-the-counter medication from pharmacies or use traditional home remedies for STDs rather than visiting STI treating clinics [10].

In spite of the fact that FSWs being regarded as core transmitters of STIs and HIV to the general population, previous studies undertaken around STIs in Ethiopia failed to consider this segment of the population. Hence, the present study investigated the prevalence of self-reported STI symptoms and the risk factors among FSWs working in licensed drinking establishments in Adama Town, Ethiopia.

## 2. Methods

### 2.1. Study Area and Period

A community-based cross-sectional study was conducted from May 01, 2017, to April 30, 2017, in Adama Town, Oromia National Regional State. Adama Town is found at the distance of 99 km from the capital city Addis Ababa. Based on the 2007 Census conducted by the Central Statistical Agency (CSA) of Ethiopia, the city has a total population of 220,212, of which 108,872 are men and 111,340 are women [11]. The town has a high concentration of bars, hotels, and night clubs where most female sex workers work. The majority of these facilities are located along the main Addis-Djibouti road, Adama-Asella road, and Kebele 15. In the town, there are an estimated 6,000 female sex workers and 95 licensed drinking establishments. There are also many street girls and female school youths who practice commercial sex work. Adama Town also hosts several schools and colleges with a high number of youth. Moreover, numerous industries and plantations attract many migrant workers from different parts of the country [12, 13].

### 2.2. Source and Study Population

Source population was all FSWs working in licensed drinking establishments found in Adama Town, while the study population was those FSWs who were randomly selected from the licensed drinking establishments found in Adama Town.

### 2.3. Sample Size and Sampling Procedure

The sample size was determined using a single population proportion formula, with an assumption of nonresponse rate of 10%. Since there is no similar study conducted in the area, during sample size calculation, the prevalence of self-reported STI symptoms among FSWs in Adama Town was assumed to be 50%. Absolute precision (d) and Z score for 95% CI were considered to be 5% and 1.96, respectively. Thus, the final sample size became 423.

Preliminary exploration was undertaken to figure out the number of FSWs working in each drinking establishment; for this, list of all licensed drinking establishments was required to undergo the preliminary exploration, and this registration list was obtained from the Adama Town cultural and tourism office. The registration list contained information such as establishment's address (kebeles and phone number) and types of the drinking establishments. Identification of the name of each establishment was achieved by contacting and consulting the community health agent of each respective kebeles office and voluntary FSWs working in the FGAE confidential clinic. Based on the registration list, the establishments were verified whether they are engaged in the business or not; furthermore, the number of FSWs working in each establishment was identified in the preliminary exploration. According to the preliminary exploration, there were 120 drinking establishments in the town that are engaged in the business, and the minimum number of FSWs working in an establishment was found to be four. Finally, the sample size was proportionally allocated to every drinking establishment, and at establishment level, FSWs were selected by the lottery method.

### 2.4. Data Collection Tool and Procedure

Data were collected using a standardized tool which was adopted from the similar study [14]. The tool has four sections: the first section contains sociodemographic characteristics of FSWs, the second section contains items related with FSWs' sexual behaviors, the third section contains FSWs knowledge of STDs and perception, and the fourth section was about the presence of STI-related symptoms in the past 12 months. Data were collected by a face to face interview, and voluntary FSWs working in FGAE (Family Guidance Association of Ethiopia) confidential clinics handled the data collection process. Two health professionals (BSc in public health) having previous experience in such types of studies were hired as facilitators.

### 2.5. Measurement

  Self-reported STI symptoms: they were assessed by asking FSWs whether they had experienced any of the following symptoms in the past 12 months: swelling in the groin area, genital ulcer, pain during urination, and genital discharge. FSWs were regarded as having STI-related symptoms, if they admitted to have at least one of the abovementioned symptoms.
  Alcohol before sex: alcohol use before having sex was measured via asking FSWs whether they ever drank alcohol before having sex with their clients. FSWs were considered to have used alcohol before having sex if they responded “yes” to the question “have you ever used alcohol before having sex with your clients in the past 12 months?”
  Drug use before sex: drug use before having sex was measured via asking FSWs whether they ever used drug before having sex with their clients. FSWs were considered to have used drug before having sex, if they responded “yes” to the question “have you ever used drug before having sex with your clients in the past 12 months?”
  Knowledge of STI: knowledge of STI was measured by asking FSWs 8 questions and having a Cronbach *α* of 0.76. The response for the first 6 items was “yes” (1 score)/“no” (0 score). The rest 2 items were to mention STI symptoms in women and men. If the respondent correctly mentions at least one symptom of STI in female or male, the respondent would have a score of 1. Then, scored points were dichotomized into good knowledge and poor knowledge depending on the mean score.
  Perceived susceptibility: the degree of perceived susceptibility to STIs was measured by asking respondents to rate their likelihood of contacting STIs in the near future (e.g., “how likely do you think it is that you would get an HIV or STI in the future?”). The response ranges from “very unlikely” to “very likely.” Then, the composite score was dichotomized into high and low perceived susceptibility depending on the mean score. Four items were used with a Cronbach *α* of 0.81.
  Perceived severity: the degree of perceived severity to STIs was measured by asking respondents to rate the adverse consequence resulting from having STIs (e.g., “one will lose his wellbeing if he/she has STIs?”). The response ranges from “very unlikely” to “very likely.” Then, the composite score was dichotomized into high and low perceived severity depending on the mean score. Four items were used with a Cronbach *α* of 0.73.

### 2.6. Data Processing and Analysis

Data were checked for completeness, edited, cleaned, coded, and entered into Epi Data version 3.1 and then were exported to SPSS version 20 for analysis. In the descriptive statistic, frequencies, proportion, and mean were calculated, and the results of the analysis were presented in text, tables, and graphs. Binary logistic regression was carried out to assess the association of different independent variables with the outcome variable (self-reported STI symptoms in the past 12 months). Independent variables having *P* < 0.25 on the binary logistic regression analysis were considered as candidates for the final multivariate logistic regression analysis. Multivariate logistic regression analysis was carried out to identify factors having statistically significant associations with the outcome variable.

### 2.7. Data Quality Assurance

To maintain the quality of data, the tool was prepared first in English and then translated to Amharic and Afaan Oromo languages and, finally, back to English in order to maintain its consistency. Three days training was provided for data collectors and supervisors about the objectives and process of the data collection. Before the actual data collection, pretesting was conducted in Mojo Town which is 18 kilometers from the study area. Modification was made based on the pretest. Finally, completed questionnaires were checked after collection for completeness and consistency by facilitators and the principal investigator.

## 3. Result

### 3.1. Sociodemographic Characteristics

Among 423 FSWs selected for the interview, 390 willingly responded to the interviewer-administered structured questionnaire making a response rate of 92.2%. The mean age of the respondents was 24.05 with a SD of ±4.18. Regarding their educational status, 89 (22.8%) of the FSWs completed grade 1–8 and 86 (22.1%) completed grade 12 and above. One hundred ninety-eight (50.8%) claimed to have stayed more than three years in the business. In spite of being engaged in sexual business, 58 (14.9%) of the FSWs reported that they are married, and 178 (45.6%) have a family to support (Table 1).

### 3.2. FSWs' Sexual Behavior

Majority of the FSWs (87.9%) commenced sexual activity before they turned 19 years. FSWs were asked whether they have used condom consistently when having sex with paying clients in the past 12 months, and 329 (84.4%) reported that they have so. Among FSWs having nonpaying partners in the past 12 months, only 121 (31%) used condom consistently when having sex with nonpaying partners. More than half of the FSWs (211 (54.1%)) used alcohol before having sex, and 96 (24.6%) encountered forced sex against their will (Table 2).

### 3.3. FSWs' STI Perception and Knowledge

According to the finding, majority of the FSWs had high perceived severity and high perceived susceptibility to STIs: 271 (69.5%) and 270 (69.2%), respectively. Regarding FSWs' knowledge of the common signs and symptoms of STIs, 225 (57.7%) mentioned at least one sign and symptom of STIs in women, and the commonly mentioned were as follows: abdominal pain (50.7%), genital discharge (39.7%), and foul-smelling discharge (31.8%). Concerning symptoms of STI in men, 265 (67.9%) of the FSWs mentioned at least one symptom of STI in men, and the commonly mentioned symptoms were genital discharge (51.8%) and burning pain during urination (46.4%). The overall comprehensive knowledge scores out of eight questions were calculated by summing the score of each item, and 199 (51%) of the FSWs had good knowledge of STIs, whereas the rest 191 (49%) had poor knowledge of STI.

### 3.4. Self-Reported STI Symptoms in the past 12 Months

**Figure 1:**
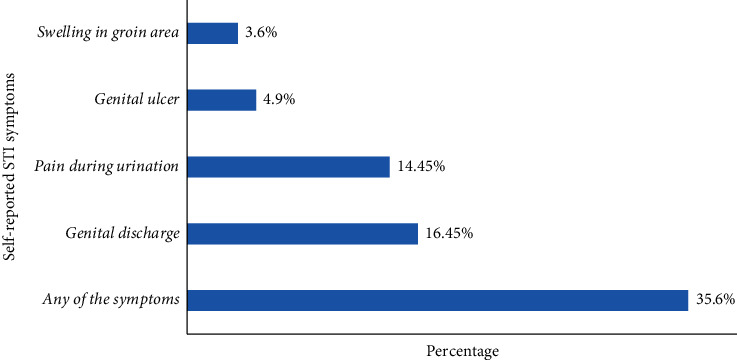
Prevalence of self-reported STI symptoms among FSWs working in licensed drinking establishments of Adama Town, Oromia regional state, Ethiopia, March 2017 (*N* = 390).

In this study, 139 (35.6%) of the FSWs admitted to have experienced at least one of the STI-related symptoms in the past 12 months. Genital discharge (16.4%) and pain during urination (14.4%) were the most common symptoms (Figure 1). FSWs with STI symptoms were asked whether they sought care for their symptoms, and all of them reported that they had. Regarding the place where they sought care, only 46.4% of the FSWs sought care from recommended health facilities (government hospital, private clinics, and NGO clinics); nevertheless, 40.6% of the FSWs bought over-the-counter medications from pharmacies and drug stores, and the rest 13% went for traditional remedies.

### 3.5. Factors Associated with Self-Reported STI Symptoms

Bivariate logistic regression was carried out to detect the discrete influence of sociodemographic characteristics, sexual behaviors, knowledge of STIs, and perceptions of STIs on the likelihood of experiencing STI-related symptoms. Accordingly, fourteen variables were discretely entered into the bivariate logistic regression, and eventually, eight variables became candidates for the final multivariate logistic regression, yielding a *P* value of ≤ 0.2. However, in the final multivariate logistic regression, only four variables, namely, duration of sex work, condom use with nonpaying clients, alcohol drinking before sex, and knowledge of STIs, were significantly associated with the likelihood of experiencing STI-related symptoms in the past 12 months (Table 3).

FSWs who stayed in the business (sex work) for more than three years were two times more likely to experience STI-related symptoms as compared to those FSWs staying in the business for less than 3 years (AOR = 2.27, 95% CI: 1.26, 4.08). FSWs using condom inconsistently with nonpaying clients were 5 times more likely to experience STI-related symptoms as compared to those who use condom consistently with their nonpaying clients (AOR = 5.43, 95% CI: 2.73, 10.80). Again, FSWs who drink alcohol before having sex were two times more likely to experience STI-related symptoms when compared with those who do not (AOR = 2.41, 95% CI: 1.35, 4.30). Lastly, FSWs having poor knowledge of STI were two times more likely to experience STI-related symptoms than those with good knowledge of STI (AOR = 2.44, 95% CI: 1.31, 4.54).

## 4. Discussion

The present study was intended to assess the prevalence of self-reported STI symptoms and their risk factors among FSWs working in licensed drinking establishments found in Adama Town. The study established that 35.6%, 95% CI (33.8%–37.4%) of the FSWs, had experienced at least one of the STI-related symptoms in the past 12 months, and similarly, the study conducted in the Republic of Korea evidenced that 38.2% of the FSWs had ever acquired at least one STD [14]. Nevertheless, this finding is much lower when compared with related studies conducted in Addis Ababa, Ethiopia (47.9%), and Goa, India (57.2%) [15, 16]. Deployment of different techniques of measuring STD among FSWs could be the reason for the discrepancy in the above finding; for instance, the study conducted in Goa, India, considered biological samples to investigate STDs among FSWs. Given the asymptomatic nature of STIs, higher prevalence could be associated with the use of laboratory investigations to detect STD among FSWs.

In this study, FSWs had a higher tendency not to use condom consistently when having sex with their nonpaying clients compared with their paying clients. This claim was confirmed by other studies undertaken in Finote Selam Town, Adama Town, Mombasa, Kenya, and Gambia [17–20]. This might imply that FSWs trust their intimate nonpaying partners and indulge in unprotected sex. Regarding alcohol consumption before sex, more than half (54.1%) of the FSWs reported that they drink alcohol before having sex, and this finding is much higher than others studies done in Finote Selam Town (46%), Philippines (19%), and China (29.4%) [17, 20, 21]. The discrepancy in this finding could be due to the study setting difference, in which the present study focused only on those FSWs working in drinking establishments (night clubs, bars, groceries, and hotels). Hence, establishment-based FSWs have a better access to alcohol than street- and home-based sex workers.

The study revealed that STI-related symptoms were more common among FSWs who reported inconsistent condom use with nonpaying clients. This was substantiated by other similar studies [16, 17]. Evidences suggest that alcohol use before sex precludes the use of condom since intoxicated FSWs are in compromised position to negotiate condom use with their clients, thereby their chance of contacting STIs increases [21–23]. Similarly, the current study revealed high STI-related symptoms among FSWs who drink alcohol before engaging in sexual intercourse.

The odds of having STI-related symptoms were higher among FSWs who stayed in the business for a long time (>3 years), and this finding is incongruent with the study done in Philippines and Mexico City [21, 24]. It could be argued that given the harsh environment that FSWs are working in, staying in the business (sex work) for several years makes FSWs to despair, feel less concerned for their own health, and engage in risky sexual behaviors.

Lastly, those FSWs who demonstrated a lower knowledge of STIs had a higher likelihood of having STI-related symptoms, and a similar claim was also suggested by the study in Goa, India [16]. FSWs often lack basic knowledge about STI prevention and let alone awareness that STI may be present even in asymptomatic individuals. Hence, not knowing the asymptomatic nature of STIs, FSWs might not engage in periodic screening and treatment of STIs.

The current study identified several factors predicting STIs among establishment-based FSWs. Hence, the finding of this study will have a huge public-health significance in mitigating the current burden of STIs/HIV among establishment-based FSWs, and based on this finding, we suggest the need for public health intervention programs focusing on promoting condom use with every client including with nonpaying/regular partners, improving FSWs' knowledge of STIs, and decreasing alcohol intake before sex. We also suggest special attention to be provided for those FSWs who had stayed longer in the business.

## 5. Conclusion

Self-reported STI symptoms among FSWs in Adama Town are relatively high when compared with previous studies. Inconsistent condom usage with a nonpaying client, alcohol use before sex, longer duration of sex work (>3 years), and having poor knowledge of STIs where found to have significant and independent association with the likelihood of experiencing STI-related symptoms among FSWs. Thus, targeted STI prevention programs focused at FSWs need to consider the above determinants in designing strategies and program aimed at mitigating the burden.

## 6. Limitations

The finding of this study has to been seen in light of some limitations. There might be a possibility of social desirability bias as data collectors were from FGAE confidential clinics; the response may be biased towards more positive answers and recall bias as participants were asked to recall their 12 months experiences. Another important limitation is the cross-sectional nature of the study which means that it is impossible to determine causality.

## Figures and Tables

**Table 1:** Sociodemographic characteristics of FSWs working in licensed drinking establishments in Adama Town, Oromia regional state, Ethiopia, March 2017 (*N* = 390).

Variables	Classifications	Frequency	Percent
Age	≥20	215	55.1
	20–39	163	41.8
	≤40	12	3.1

Educational status	Unable to read and write	52	13.3
	Read and write	47	12.1
	Grade 1–8	89	22.8
	Grade 10–12	94	24.1
	Above grade 12	86	22.1

Marital status	Single	332	85.1
	Married	58	14.9

Religion	Orthodox Christian	137	35.1
	Muslim	117	30.0
	Protestant Christian	72	18.5
	Catholic Christian	49	12.6
	Others	15	3.8

Any family to support	Yes	178	45.6
	No	212	54.4

Duration of sex work	1–3 years	192	49.2
	>3	198	50.8

**Table 2:** Sexual behavior of FSWs working in licensed drinking establishments of Adama Town, Oromia regional state, Ethiopia, March 2017 (*N* = 390).

Variables	Classifications	Frequency	Percent
Age at the first sexual intercourse	<10 years	13	3.3
	10–15 years	174	44.6
	16–18 years	152	40
	Above 18 years	51	13.1

Have nonpaying sexual partner	Yes	272	69.7
	No	118	30.3

Number of nonpaying partners	One	65	16.7
	More than one	207	53.1

Consistent condom use with nonpaying partners	Yes	121	31
	No	269	69

Consistent condom use with paying clients	Yes	329	84.4
	No	61	15.6

Alcohol use before sex	Yes	211	54.1
	No	179	45.9

Drug use	Yes	123	31.5
	No	276	71.5

Experienced forced sex in the past 12 months	Yes	96	24.6
	No	294	75.4

**Table 3:** Factors associated with self-reported STI symptoms among FSWs working in licensed drinking establishments of Adama Town, Oromia regional state, Ethiopia, March 2017 (*N* = 390).

Variables	STI symptoms		COR (95% CI)	AOR (95% CI)
	Yes (%)	No (%)		
*Age*				
≥20 year	81 (37.7%)	81 (37.7%)	1.00	1.00
20–39 year	50 (30.7%)	50 (30.7%)	(0.45, 1.12)	0.82 (0.45, 1.50)
≤40 year	81 (37.7%)	8 (66.7%)	(0.96, 11.33)	6.77 (0.98, 32.62)

*Duration of sex work*				
1–3 years	46 (24.0%)	146 (76.0%)	1.00	1.00
≥3 years	93 (47.0%)	105 (53.0%)	2.81 (1.82, 4.33)	**2.27 (1.26, 4.08)**

*Consistent condom use with nonpaying clients*				
Yes	38 (31.4%)	83 (68.6%)	5.02 (2.76, 9.11)	**5.43 (2.73, 10.80)**
No	101 (37.5%)	168 (62.5%)	1.00	1.00

*Experienced forced sex*				
Yes	102 (34.7%)	192 (65.3%)	1.00	1.00
No	37 (38.5%)	59 (61.5%)	1.18 (0.73, 1.90)	0.42 (0.20, 0.91)

*Alcohol use before sex*				
Yes	40 (22.3%)	139 (77.7%)	1.00	1.00
No	99 (46.9%)	112 (53.1%)	3.07 (91.97, 4.78)	**2.41 (1.35, 4.30)**

*Perceived susceptibility*				
Low	37 (31.1%)	82 (68.9%)	1.00	1.00
High	102 (37.6%)	169 (62.4%)	1.33 (0.84, 2.18)	1.22 (0.58, 2.58)

*Perceived severity*				
Low	34 (28.3%)	86 (71.7%)	1.00	1.00
High	105 (38.9%)	165 (61.1%)	1.61 (1.01, 2.56)	1.82 (0.94, 3.53)

*Knowledge of STI*				
Good	85 (44.5%)	106 (55.5%)	2.15 (1.41, 3.28)	**2.44 (1.31, 4.54)**
Poor	54 (27.1%)	145 (72.9%)	1.00	1.00

## Data Availability

The datasets collected and analyzed for the current study are available from the corresponding author and can be obtained upon reasonable request.

## Abbreviations

AOR: Adjusted odd ratio
CI: Confidence interval
CSA: Central statistical agency
FGAE: Family Guidance Association of Ethiopia
FSWs: Female sex workers
SPSS: Statistical package for social scientists
STIs: Sexually transmitted diseases.

## References

[1] Minichiello V., Rahman S., Hussain R. (2013). Epidemiology of sexually transmitted infections in global indigenous populations: data availability and gaps. *International Journal of STD & AIDS*.

[2] WHO (2018). *Report on Global Sexually Transmitted Infection Surveillance*.

[3] Pankhurst R. (2017). The history of prostitution in Ethiopia. *Journal of Ethiopian Studies*.

[4] (1994).

[5] Family Health International (FHI) Mapping and census of female sex workers in Addis Ababa, Ethiopia, 2002: https://www.who.int/hiv/topics/vct/sw_toolkit/mapping_census_female_sex_workers_ethiopia.pdf?u=1

[6] SWAZILAND. Has Swaziland turned the corner in the fight against AIDS? Irin News. 2006 http://www.irinnews.org/report.aspx?reportid=62330

[7] USAID Region Report (2005). *Economic deprivation of African women*.

[8] Shannon K., Bright V., Gibson K., Tyndall M. W. (2007). Sexual and drug-related vulnerabilities for HIV infection among women engaged in survival sex work in vancouver, Canada. *Canadian Journal of Public Health*.

[9] Liu S.-H., Srikrishnan A. K., Zelaya C. E., Solomon S., Celentano D. D., Sherman S. G. (2011). Measuring perceived stigma in female sex workers in Chennai, India. *AIDS Care*.

[10] (2019). http://www.chp.ac.za/PolicyBriefs/Documents/Differ%20policy%20brief%20FINAL.pdf%20.

[11] (2018). https://microdata.worldbank.org/index.php/catalog/2747.

[12] The Federal Democratic Republic of Ethopia (2018). *Central Statistical Agency, Statistical Report on the 2012 Urban Employment Unemployment 2012*.

[13] Mela Research (2014). Know your HIV epidemic/know your HIV response (KYE/KYR) synthesis in Oromia, Ethiopia. http://www.melaresearch.com/Documents/melarep14.pdf.

[14] Jung M. (2019). Risk factors of sexually transmitted infections among female sex workers in Republic of Korea. *BMC Public Health*.

[15] Desta S., Feleke W., Yusuf M. (1990). Prevalence of STD and STD related risk factor in sex workers of Addis Ababa. *The Ethiopian Journal of Health Development*.

[16] Shahmanesh M., Cowan F., Wayal S., Copas A., Patel V., Mabey D. (2009). The burden and determinants of HIV and sexually transmitted infections in a population-based sample of female sex workers in Goa, India. *Sexually Transmitted Infections*.

[17] Anteneh Z. A., Anteneh Y. A. A., Agumas Y. A., Tarekegn M. (2017). Sexually transmitted diseases among female commercial sex workers in Finote Selam town, northwest Ethiopia: a community-based cross-sectional study. *HIV/AIDS-Research and Palliative Care*.

[18] Mooney A., Kidanu A., Bradley H. M., Kumoji E. K., Kennedy C. E., Kerrigan D. (2013). Work-related violence and inconsistent condom use with non-paying partners among female sex workers in Adama City, Ethiopia. *BMC Public Health*.

[19] Parcesepe A. M., L’Engle K. L., Martin S. L. (2016). Early sex work initiation and condom use among alcohol-using female sex workers in Mombasa, Kenya: a cross-sectional analysis. *Sexually Transmitted Infections*.

[20] Bo W., Xiaoming L., Bonita S., Lei Z., Xiaoyi F. (2010). Alcohol use, unprotected sex, and sexually transmitted infections among female sex workers in China. *Sexually Transmitted Diseases*.

[21] Chiao C., Morisky D. E., Rosenberg R., Ksobiech K., Malow R. (2006). The relationship between HIV/Sexually Transmitted Infection risk and alcohol use during commercial sex episodes: results from the study of female commercial sex workers in the Philippines. *Substance Use & Misuse*.

[22] Maher L., Mooney-Somers J., Phlong P. (2013). Condom negotiation across different relationship types by young women engaged in sex work in Phnom Penh, Cambodia. *Global Public Health*.

[23] Jung M. (2012). Sexual, behavioral, and social characteristics of female sex workers and their risk of sexually transmitted infections: in South Korea. *Sexuality and Disability*.

[24] Uribe-Salas F., Hernandez-Avila M., Juarez-Figueroa L., Conde-Glez C. J., Uribe-Zúñiga P (1999). Risk factors for herpes simplex virus type 2 infection among female commercial sex workers in Mexico City. *International Journal of STD & AIDS*.

